# A 3D-Printed Micro-Optofluidic Chamber for Fluid Characterization and Microparticle Velocity Detection

**DOI:** 10.3390/mi14112115

**Published:** 2023-11-18

**Authors:** Emanuela Cutuli, Dario Sanalitro, Giovanna Stella, Lorena Saitta, Maide Bucolo

**Affiliations:** 1 Department of Electrical Electronic and Computer Science Engineering, University of Catania, Via Santa Sofia 64, 95125 Catania, Italy; dario.sanalitro@unict.it (D.S.); giovanna.stella@phd.unict.it (G.S.); maide.bucolo@unict.it (M.B.); 2 Department of Civil Engineering and Architecture, University of Catania, Via Santa Sofia 64, 95125 Catania, Italy; lorena.saitta@phd.unict.it

**Keywords:** microfluidics, micro-optics, signal processing, lab on a chip, DPIV, dual-slit

## Abstract

This work proposes a multi-objective polydimethylsiloxane (PDMS) micro-optofluidic (MoF) device suitably designed and manufactured through a 3D-printed-based master–slave approach. It exploits optical detection techniques to characterize immiscible fluids or microparticles in suspension inside a compartment specifically designed at the core of the device referred to as the *MoF chamber*. In addition, we show our novel, fast, and cost-effective methodology, dual-slit particle signal velocimetry (DPSV), for fluids and microparticle velocity detection. Different from the standard state-of-the-art approaches, the methodology focuses on signal processing rather than image processing. This alternative has several advantages, including the ability to circumvent the requirement of complex and extensive setups and cost reduction. Additionally, its rapid processing speed allows for real-time sample manipulations in ongoing image-based analyses. For our specific design, optical signals have been detected from the micro-optics components placed in two slots designed *ad hoc* in the device. To show the devices’ multipurpose capabilities, the device has been tested with fluids of various colors and densities and the inclusion of synthetic microparticles. Additionally, several experiments have been conducted to prove the effectiveness of the DPSV approach in estimating microparticle velocities. A digital particle image velocimetry (DPIV)-based approach has been used as a baseline against which the outcomes of our methods have been evaluated. The combination of the suitability of the micro-optical components for integration, along with the *MoF chamber* device and the DPSV approach, demonstrates a proof of concept towards the challenge of real-time total-on-chip analysis.

## 1. Introduction

The design of highly complex microsystems for lab-on-a-chip (LOC) applications exhibits the potential to transform the analyses of biological [1,2,3,4,5,6], chemical [1,6,7], and medical [8,9,10] small fluid expenditure. Low-cost [1,10,11] and miniaturized [3,12] systems are swiftly becoming deployable [11], disposable [4], and automated [1] solutions. These designs require a careful selection of methodologies and technologies for biological and chemical fluid detection and control. Among all the proposed state-of-the-art solutions, several detection methods are used in the LOC field for monitoring and analyzing the fluids and particles [13,14] as: optical [15,16], electrochemical [17,18,19], flow cytometry [20], micro-wave [21], and so on. In this context, the optical technologies, including microscopes, lasers, spectrophotometers, charge-coupled devices (CCDs) [22], and photomultiplier tubes (PMTs) [23] are perfectly suitable for being integrated into microfluidic applications, as they are non-invasive technologies [24].

Other methodologies, heavily relying on images, such as particle image velocimetry (PIV) [25,26], particle tracking velocimetry (PTV) [27,28], X-ray imaging [29], nuclear magnetic resonance imaging [30], infrared imaging [31], and optical Doppler tomographic velocity imaging [32] are all used to investigate and quantitatively characterize fluid flow inside microchannels. Together, optical tools and imaging methods are the fundamental components of current microfluidic systems. Multiple fields offer viable alternatives to conventional optical detection methodologies. Micro-optofluidics, a branch of microfluidics, focuses on the integration of optical and fluidic components into microscale systems with the ultimate goal of developing LOC devices that can manipulate both light and fluids at the microscale without the requirement of a bulky and costly equipment [29,33,34,35,36]. Individual optical components, such as photodiodes, waveguides, lenses, and optical fibers, have been successfully integrated into microfluidic systems to investigate small volumes of fluids flowing inside microdevices [33,37,38,39,40,41].

However, despite these advances, the state-of-the-art approaches exhibit several limitations: (i) the challenge of miniaturization in the development of low-cost and portable detection devices remains unresolved; (ii) existing investigation methodologies require complex and expensive setups; (iii) these methodologies also require high computational costs and take significant time to perform the analyses; and (iv) additionally, they lack the capability to estimate high microparticle velocities.

In this work, we aim to address the mentioned limitations by presenting a multi-objective micro-optofluidic (MoF) device suitably designed and manufactured through a 3D-printed-based master–slave approach which integrates optical components within a microfluidic chip. The system relies on the light absorption phenomenon to perform several tasks. It can characterize immiscible fluids or microparticles in suspension. Such fluids are injected into a compartment specifically designed at the device core, referred to as the *MoF chamber*.

In addition, thanks to the specific proposed chip design, the study presents a novel and image-free application of the cross-correlation methodology for fluid and microparticle velocity estimation. Our approach, referred to as dual-slit particle signal velocimetry (DPSV), enables fluid velocity estimation through the computation of the correlation between optical signals obtained from specifically designed micro-optic components positioned in two slots intentionally made inside the device. Being signal-based and benefiting from its high operational frequency, the approach can be used in conjunction with image processing methodologies for real-time sample manipulations (or process control) [42,43]. Compared to the most used state-of-the-art techniques, the proposed method is label-free, non-invasive, cost-effective, and computationally efficient. All these attributes contribute to its potential use in total-on-chip real-time analyses.

In fact, the proposed design offers ways of delving into studies concerning various cell cultures aspects such as cell growth and death, complex cell interactions, and the impact of drugs on cellular behavior. The possibility of parallelizing the image acquisition through CCD images and signal acquisition via photodetector can pave the way for simultaneous sample manipulation via signal processing and process spatio-temporal monitoring with imaging techniques. In particular, the validity of the image processing approach has been demonstrated in for the spatio-temporal RBC investigation in micro-vessels [44] and microchannels [45,46] and the integration of optical signal detection has been showed for particle concentrations [47].

To show the device’s multipurpose capabilities, fluids of various colors and densities were introduced in the *MoF chamber*. Additionally, silica beads were placed within the device to prove how well our DPSV approach estimates the microparticle velocities. The results have been compared with the estimated silica beads velocities obtained through the DPIV-based approach presented in [47]. Such an approach has been used as a baseline against which the outcomes of our proposed method have been evaluated. As a result, the performed comparison confirms the feasibility of the proposed methodology other than its benefits of simplicity, low cost, and nearly zero computational time.

In this context, our contributions are as follows: (i) Design, implementation, and realization of a 3D-printed-based MoF device which integrates micro-optics and microfluidic components. Such a device is suitable to perform fluid characterization. (ii) Particle velocity estimation through a label-free, non-invasive, cost-effective, and minimally computationally demanding methodology which relies on cross-correlation computation between optical signals.

The paper is organized as follows. Section 2 describes the micro-optofluidic device working principle, showing the ray-tracing simulations to optimize the device geometry. The *MoF chamber* 3D-printing-based manufacturing approach is also presented. Following this, the novel proposed methodology for microparticles velocity detection is presented in Section 3. In Section 4 the experimental setups used for the fluids characterization and the microparticles velocity detection are described. The main results are summarized in Section 5 and Section 6. In Section 5, the device’s capability to differentiate between fluids of various colors and natures is examined. Then, in Section 6, the *MoF chamber* and DPSV method are tested for the detection of microparticles and their velocity in different experimental conditions. The good performance of the system was confirmed by comparison with computational fluid dynamic (CFD) analysis and the DPIV-based methodology.

## 2. Micro-Optofluidic Chamber: Working Principle, Design, and Manufacturing

### 2.1. Device Working Principle

**Figure 1:**
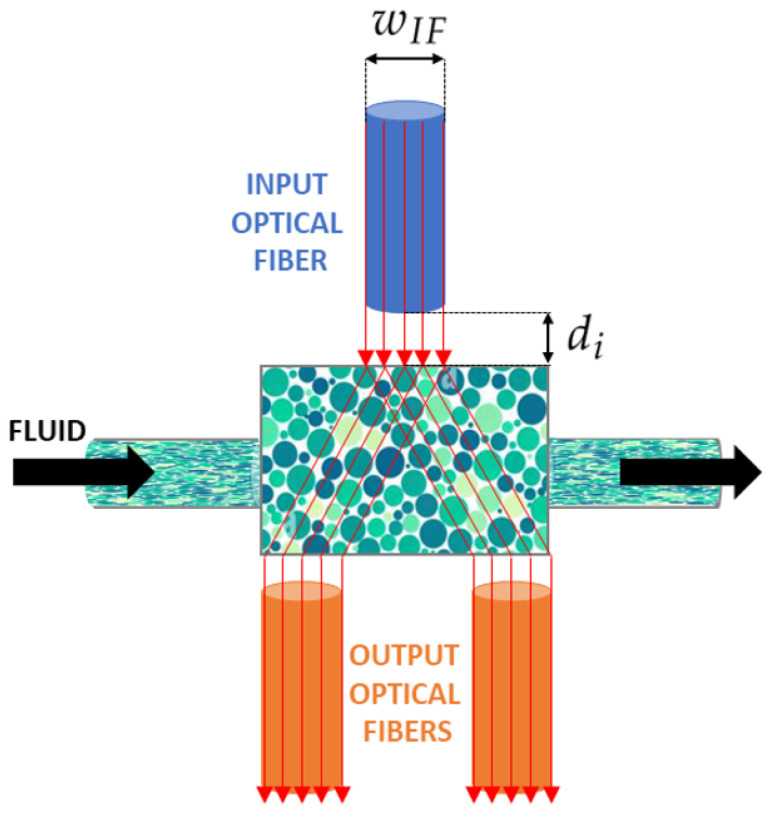
Working principle of the micro-optofluidic device: $w_{IF}$ and $d_{i}$ represent the input fiber insertion width and the *MoF chamber* input fiber inter-distance, respectively.

In this work, we present a polydimethylsiloxane (PDMS) micro-optofluidic (MoF) device that uses the light absorption phenomenon to monitor two-phase microfluidic processes. The term *two-phase processes* refers to two immiscible fluids, one dispersed in the other, which circulate within the same microsystem [48]. They are generally formed by immiscible liquid–liquid [49,50,51,52], gas–liquid [53,54], and microparticles suspended in a liquid [47,55]. In particular, the device aims to characterize immiscible fluids or microparticles in suspension inside a compartment specifically designed at the core of a microfluidic channel, referred to as the *MoF chamber*. Particularly, it is designed to be a small total-on-chip device for the label-free characterization of cell populations subjected to hydrodynamic stimuli and their velocity detection exploiting micro-optical components. As shown in Figure 1, the fluid, introduced from the inlet, travels through a microchannel with a cross section equal to 400 (μm) × 400 (μm), towards the *MoF chamber*, which represents the device’s portion where the fluid sample is examined. The outlet allows the fluid release. Additionally, the device includes three micrometric slots orthogonally placed with respect to the *MoF chamber*. One (in blue in Figure 1) is designated for the optical actuation system and contains the input optical fiber (*IF*). It is $w_{IF}$ wide and $d_{i}$ distant from the *MoF chamber*. The other two slots, on the opposite side (in orange in Figure 1), constitute the optical detection system with two slots of 400 (μm) × 400 (μm) cross sections for the output optical fibers, $OF_{1}$ and $OF_{2}$, respectively. The device’s working principle is described as follows. A light beam is generated by the light source produced by the actuation system and directed via *IF*. The beam then travels through the *MoF chamber* which contains the solution of interest. The outgoing beam is collected by $OF_{1}$ and $OF_{2}$ and sent to the detecting devices. Due to the fluid’s absorption, the exiting light beams will have less intensity than the entering light beam. Such a difference is then used to extrapolate the properties of the fluids under investigation.

### 2.2. Ray-Tracing Simulations and Geometry Optimization

Since the interplay between light and fluids traveling through the system is crucial to the device’s realization, ray-tracing simulations were run using TracePro. The aim was to determine how different geometrical characteristics can affect the devices’ performances. In particular, three distinct devices D-1, D-2, and D-3 were designed and then evaluated. Their design choices are the results of different combinations of *MoF chamber width* and *height*, $w_{IF}$ and $d_{i}$ values, with the goal of optimizing the *MoF chamber* lighting. In contrast, the microchannel dimensions, the $OF_{1}$ and $OF_{2}$ insertion sections, their inter-distance (5 mm) and the one from the *MoF chamber* (5 mm) were fixed at constant values to guarantee device fabrication stability and to maximize the optical acquisition output according on the study proposed in [55].

**Figure 2:**
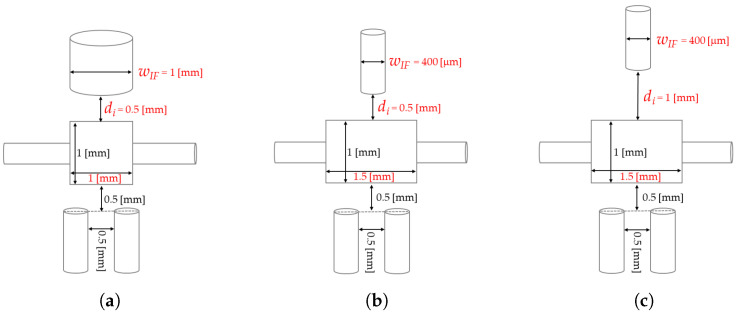
(**a**–**c**) Detailed geometric dimensions of prototypes D-1, D-2, and D-3, respectively.

Table 1 summarizes the dimensions of the three prototypes, which are depicted in Figure 2a–c. In D-1 the *MoF chamber* dimension is 1 mm × 1 mm × 1 mm, the diameter of *IF*’s insertion is 1 mm and the distance between the latter and the *MoF chamber* is 0.5 mm. In D-2 the *MoF chamber* dimensions were changed in 1 mm × 1.5 mm × 0.4 mm, the diameter of *IF* reduced at 0.4 mm and its distance from the *MoF chamber* was maintained at 0.5 mm. Finally, in D-3 the *MoF chamber* dimensions and the diameter of *IF* were maintained, while their inter-distance was increased at 1 mm to maximize the amount of detected light.

Two set of simulations were performed. They consist of introducing 200 rays at a power P = 1 (mW) into the *IF* of each device. The numerical aperture of the *IF* was set with a circular shape and a semi-diameter equal to 10${}^{\circ }$, while the core size of the optical fibers was defined equal to 369 μm.

**Figure 3:**
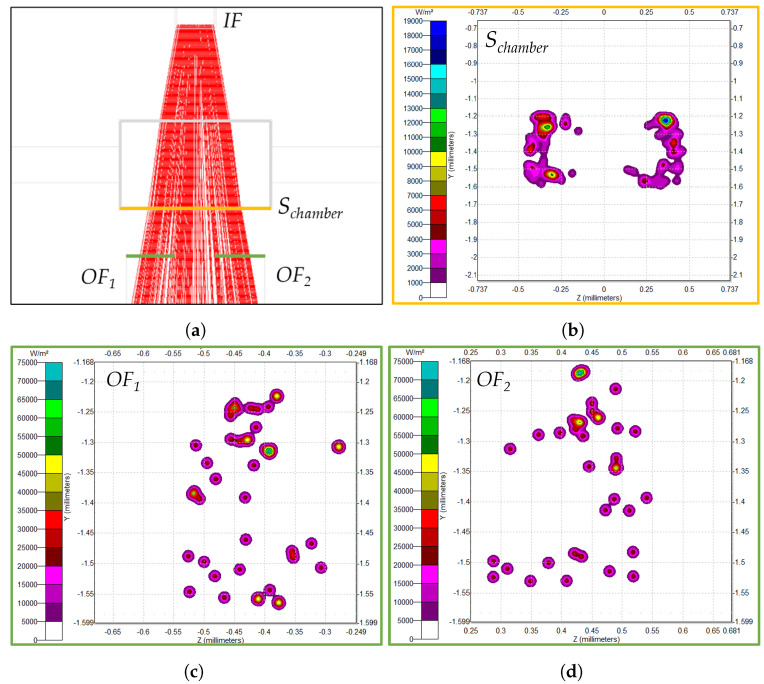
TracePro simulation results along the sections under investigation for D-3 assuming an *MoF chamber* filled with water (Sim-2). (**a**) Incident ray path top view. (**b**–**d**) Irradiance maps of the incident ray distributions along $S_{chamber}$, $OF_{1}$, and $OF_{2}$.

The first set assumes that the *MoF chamber* is filled with air (Sim-1), and the second one assumes that the *MoF chamber* is filled with water (Sim-2). Irradiance maps were produced to determine the number of incoming rays perceived by the optical fibers and evaluate the quality of the detection. Figure 3 summarizes the main outcomes of the simulations. In detail, Figure 3a shows the incident rays’ path top view for D-3, assuming an *MoF chamber* filled with water (Sim-2), moving from IF to $OF_{1}$ and $OF_{2}$. The number of incident rays were evaluated in different surfaces, i.e., the *MoF chamber* surface ($S_{chamber}$) from which the rays come out (in yellow in Figure 3a) and the input surfaces of the two output optical fibers (in green in Figure 3a). Their irradiance maps are displayed in Figure 3b–d.

By focusing on Figure 3b, it is evident that the incident ray distribution on $S_{chamber}$ does not have a circular shape. This is related to the *height* of the *MoF chamber*, coinciding with that of the *IF* insertion, and equal to 400 μm. As a result, the rays that diverge from *IF* on the basis of the fact that the numerical apertures do not traverse the *MoF chamber* surface in the *height* dimension because they surpass it.

**Figure 4:**
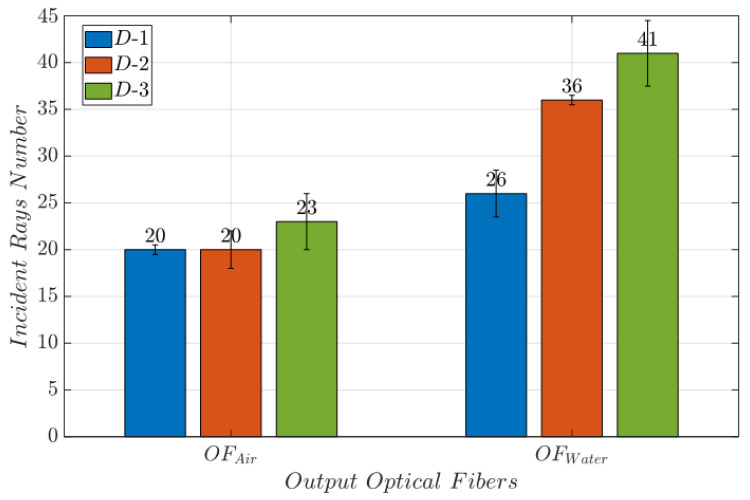
Bar plot reporting the average number of incident rays on the surface of interest at the water (Sim-2) passage. The error bars represent the uncertainty associated to the measurements when it is conducted by means of $OF_{1}$ rather than $OF_{2}$.

By comparing the results obtained from (Sim-1) and (Sim-2), the number of incident rays in $S_{chamber}$ is higher (96 rays) when the *MoF chamber* is filled with water than with air (56 rays). Notably, the higher number of incident rays for water than for air can be attributed to the change in the medium. When light transitions from PDMS to water, the refractive indexes (PDMS = 1.412, water = 1.3) are closely aligned, almost creating a seamless transition between the materials. Conversely, when light travels from PDSM to air (air = 1), there is a significant shift in the refractive index which causes the incident rays to deviate more, resulting in a decrease in the number of observable rays. Further evidence is represented by the bar plots in Figure 4. They report the average number of incident rays on the surface of interest at the air (Sim-1) and water (Sim-2) passage for D-1, D-2, and D-3. The error bars represent the level of uncertainty in measurements conducted with $OF_{1}$. This uncertainty is associated with a simulated light source.

Therefore, it is determined that when the $d_{i}$ is larger and $w_{IF}$ is lower, more incoming rays are sensed with the output optical fiber. D-3 has these characteristics and outperforms D-1 and D-2. Indeed, only a loss in the incident rays’ rate equal to 15% and 16%, for water and air, respectively, was detected from the input surfaces of the output optical fibers when compared to the *MoF chamber* surface.

### 2.3. Device Manufacturing

The MoF device is realized in PDMS employing a master–slave approach based on an inkjet 3D printing technology. The method proposed here, which falls within the category of soft lithography manufacturing methods, allowed us to overcome some well-known issues related to standard master–slave micro-fabrication approaches commonly used to fabricate microfluidic devices, such as photolithography [56,57]. Indeed, the latter method involves (i) expensive fabrication costs; (ii) complexity of processing, since the mold fabrication requires several time-consuming steps where a careful control of various parameters should be performed [58,59]; (iii) clean-room requirements to minimize dust and particle contamination; (iv) expensive equipment; and (v) the handling of hazardous chemicals, which may cause serious environmental impact if proper disposal methods are not used. Conversely, the master–slave approach proposed here relies on mold fabrication via inkjet 3D printing which is an easy, low-cost, and one-step process without directly handling hazardous chemicals. In fact, the final cost for the 3D printed mold, evaluated by using the cost model already proposed by the authors [60,61], is of about 11 € per part. Thus, the mold (acting as master in our approach) is used as tool to realize the final MoF device in PDMS.

**Figure 5:**
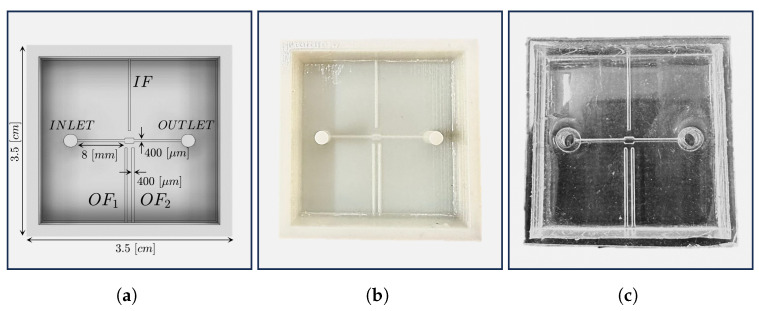
(**a**) Upper view of the *MoF chamber* CAD design. (**b**) 3D-printed master of the *MoF chamber*. (**c**) PDMS *MoF chamber*.

The realization consists of several essential steps. Firstly, the device’s mold was designed (see Figure 5a) using the Autodesk^®^ Fusion 360 (v.2.0.17721) software and an STL file was generated. The latter was processed through the proprietary software Objet Studio^TM^ (v.9.2.11.6825) (Stratasys, Los Angeles, CA, USA) with the aim to carry out the building preparation, i.e., to perform the slicing procedure and obtain the G-code instructions for the 3D printer. Next, the mold creation started, and this step was accomplished using a PolyJet 3D printer Stratasys Objet260 Connex 1 (Stratasys, Los Angeles, CA, USA). Its working principle relies on the jetting of small droplets of liquid photopolymer ink (Vero PureWhite RDG837, OVERMACH S.p.A, Parma, Italy) on the build tray. They are instantaneously photocured (i.e., solidified) using irradiation with a light source, a UV lamp placed on the printhead itself. The building process of the part followed a layer-by-layer deposition of the photocurable resin protocol. To guarantee the part’s adhesion on the build tray, a proper support material (FullCure705, OVERMACH S.p.A, Parma, Italy) was used. Similarly to the model material, it was deposited through injection on the build tray and photocured by means of the UV lamp as well. Then, it was washed out through water jetting, in line with the Stratasys post-printing process guide.

The final 3D-printed mold is shown in Figure 5b. Once the 3D printing, i.e., the building procedure of mold was accomplished, its surface was subjected to an ultraviolet (UV) treatment at 35 ${}^{\circ }$C for 1 h with the aim of further avoiding the possibility of leaving surface zones not fully photocured, thus compromising the final superficial finish of the PDMS device poured within the mold.

Then, the PDMS mixture was obtained by mixing the silicone elastomer base and the curing agent (Sylgard 184 elastomer kit, Dow Corning) according to the mixing ratio of (10:1) for the device layer (micro-systems) and (5:1) for the bulk cover layer. A degassing procedure was performed in a desiccator under vacuum conditions to remove bubbles generated during the mixing phase. Next, the mixture was poured over the master mold and cured at room temperature for 48 h. In the end, the manufactured PDMS device was finally demolded from the mold and further bounded with a bulk cover of 0.5 mm thickness using a reversible bounding procedure. The final assembled device is shown in Figure 5c.

According to the quality monitoring analysis previously conducted by the authors [61], accounting for the selected inkjet 3D printing process accuracy required, during the mold design phase an offset (bias setup) of 200 μ was set with respect to the nominal dimensions, which were fixed by using the ray-tracing simulations in the Section 2.2. In this way, thanks to a proper design for additive manufacturing (DfAM) strategy, it was possible to obtain the desired actual values for the fundamental size of the *MoF chamber*. Moreover, the measured average surface roughness value of the final device made of PDMS in [62], which strictly depends on the 3D printed mold’s surface roughness, is equal to 0.763 nm. Thus, with the relative roughness for the inlet feed channel equal to 0.0004%, no instability of the flow inside the channel correlated to the surface roughness for the manufactured device is expected. For this reason, mold manufacturing via the selected inkjet 3D printing technique can guarantee a stable flow inside the MoF microchannel and the *MoF chamber*. For comparison with similar studies presented in literature, Taylor et al. [63] proved that even though relative surface roughness values lower than 5% in macrochannels can be neglected, since they do not affect the performance of the fluid flow, it is not true for channels with micrometric size. However, Dai and Li [64] pointed that the relative roughness critical value for moving from a smooth microchannel and a rough one is 1%. Thus, as our relative surface roughness is strongly lower than this critical value, the influence of the surface roughness on the fluid flow is negligible in our study.

## 3. Methodology

In this section, we describe our signal-based methodology for microparticle velocity estimation, specifically called *dual-slit particle signal velocimetry* (DPSV). Additionally, we review the basics of an image-based methodology which incorporates a DPIV analysis with an ad hoc post-processing procedure that allows extracting of the mean microparticle velocities over time [47]. Such an approach will serve as a baseline against which the outcomes of our proposed method will be evaluated.

### 3.1. Dual-Slit Particle Signal Velocimetry

Different from the state-of-the-art approaches, DPSV adopts signal pair comparisons rather than image pair analyses to estimate the microparticles’ velocities. As a result, it is quick, with a computation time that is 600 times less than the DPIV-based method, and well suited for real-time applications needing real-time process analysis.

**Figure 6:**
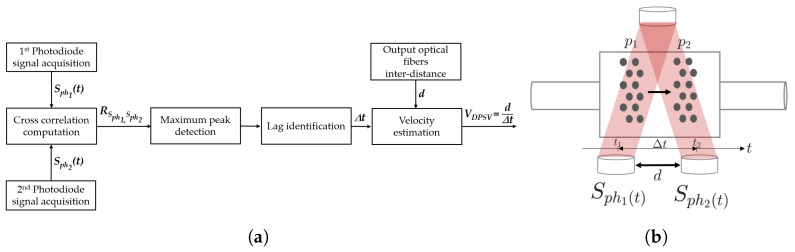
(**a**) Flow chart and (**b**) graphical representation of the dual-slit particle signal velocimetry (DPSV) methodology’s working principle.

Its working principle is summarized in the flow chart of Figure 6a and it is graphically illustrated in Figure 6b. It works as follows. It is assumed that the *MoF chamber* is filled with a sample of suspended microparticles moving in the horizontal direction from left to right. At the time instant $t_{1}$, the microparticles are in position $p_{1}$. After a time interval Δt, depending on their velocity, they reach the position $p_{2}$ at the time instant $t_{2}$. Thus, the particle–light interaction results detected using the photodiode $ph_{1}$ at $t_{1}$ are similar to those detected using photodiode $ph_{2}$ at $t_{2}$ when a Δt interval elapses. For the detected optical signals $S_{ph_{1}}\left(t\right)$ and $S_{ph_{2}}\left(t\right)$, the cross-correlation $R_{S_{ph_{1}},S_{ph_{2}}}$ between $S_{ph_{1}}\left(t\right)$ and $S_{ph_{2}}\left(t\right)$ is computed according to Equation (1). The objective is to estimate the similarity between the two signals as function of the lag in time (Δt).

(1) $$R_{S_{ph_{1}},S_{ph_{2}}}=\int_{-\infty }^{+\infty }S_{ph_{1}}\left(t\right)\;S_{ph_{2}}\left(t+\Delta t\right)\;dt$$

Operatively, as shown in [65], cross-correlations are computed by considering them as a sequence of two jointly stationary random processes $x_{n}$ and $y_{n}$, i.e.,

(2) $$R_{xy}\left(m\right)=E\left\{x_{n+m}y_{n}^{*}\right\}E\left\{x_{n}y_{n-m}^{*}\right\}$$

where −∞<n<∞, the operator ${}^{*}$ denotes complex conjugation, and *E* is the expected value operator. Therefore,

(3) $${\hat{R}}_{xy}\left(m\right)=\left\{\begin{array}{cc} \sum_{n=0}^{N-m-1}x_{n+m}y_{n}^{*} & m\geq 0\\ {\hat{R}}_{yx}^{*}\left(-m\right) & m<0 \end{array}\right.$$

After the $R_{S_{ph_{1}},S_{ph_{2}}}$ calculation, the maximum peak in the cross-correlation function is identified. This information represents the point at which the two signals being cross-correlated align most closely with each other in terms of time or lag (Δt). By knowing a priori the design parameter *d* between the two detection systems (shown in Figure 6b), the velocity microparticles’ mean value $V_{DPSV}$ can be estimated as follows:

(4) $$V_{DPSV}=\frac{d}{\Delta t}$$

The value of $V_{DPSV}$ represents the mean of the microparticle velocity used for the flow characterization.

### 3.2. DPIV-Based Algorithm

To validate our DPSV, we conduct a comparative analysis with the DPIV-based algorithm that has been previously validated and presented in [47]. Specifically, such an algorithm incorporates a DPIV analysis and an ad hoc post-processing procedure that allows extraction of the mean microparticle velocity trends over time.

As an image-based approach, it performs an evaluation of two consecutive frames of a recorded video by means of a three-pass discrete Fourier transform (DTF) in the frequency domain. As a result, time-varying velocity vector maps are obtained, one in the horizontal, and one in the vertical direction for each pair of frames.

These maps are then analyzed using an ad hoc post-processing procedure. In detail, the velocitys’ spatial distributions along the horizontal $V_{x}\left(i,j,t\right)$ and vertical $V_{y}\left(i,j,t\right)$ directions are spatially averaged to obtain a comprehensive velocity value for each spatial map per time sample *t*, where *i* and *j* refer to the region of interest’s (ROI) pixel positions. By considering the velocity values obtained from all the spatial maps, the outcome is the generation of two average velocity signals in the *x* and *y* directions, ${\bar{V}}_{x}\left(t\right)$ and ${\bar{V}}_{y}\left(t\right)$ respectively. In this work, given the forcing oscillating input direction and the predominance of the mean horizontal velocity component, the parameter estimated by following the previously described analysis will be referred to ${\bar{V}}_{x}\left(t\right)$. The information collected in ${\bar{V}}_{x}\left(t\right)$ is then used to identify the maximum velocity value $max\left({\bar{V}}_{x}\left(t\right)\right)=V_{DPIV}$ reached by the microparticles.

## 4. Experimental Setup

**Figure 7:**
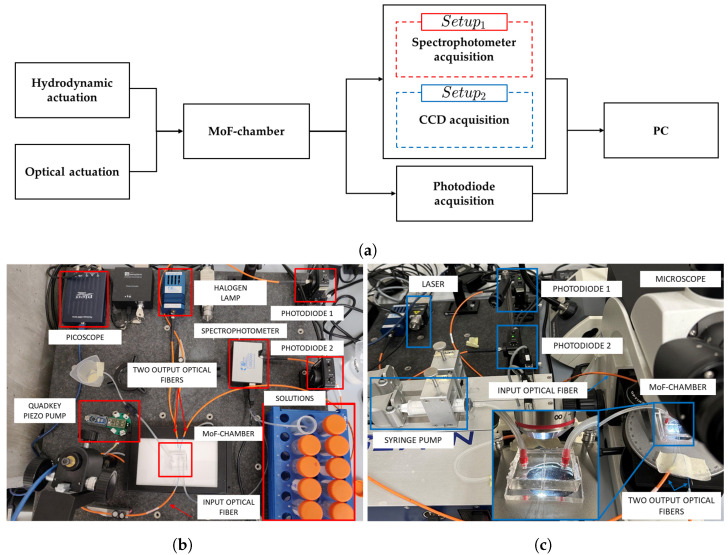
(**a**) Complete block diagram. (**b**) Real experimental $Setup_{1}$ used for the fluid detection and (**c**) $Setup_{2}$ for microparticle velocity detection.

The system used for micro-optofluidic *MoF chamber* characterization is schematically depicted in Figure 7a. In particular, it is composed of (i) the hydrodynamic actuation system for injecting the fluid sample inside the *MoF chamber*, (ii) the optical actuation system, (iii) the *MoF chamber*, (iv) a photodiode acquisition system, (v) a spectrophotometer acquisition system for $Setup_{1}$ (in red), (vi) a video acquisition system for the $Setup_{2}$ (in blue), and (vii) a PC hosting the running algorithm. From now on, it is referred to as $Setup_{1}$ for fluid detection and as $Setup_{2}$ for microparticle velocity detection. The real experimental setups are shown in Figure 7b,c.

In both scenarios, the *MoF chamber* was connected through a SMA connector to a 365 μm diameter *IF* and, in the opposite side, it was coupled with two 365 μm diameter output optical fibers, $OF_{1}$ and $OF_{2}$, one connected to the spectrophotometer and the other to the photodiode.

Specifically, in $Setup_{1}$, a continuous single-phase flow was generated by pumping fluids of different natures to the inlet of the MoF device. A piezo pump (mp6, Bartels mikrotecknik, Dortmund, Germany) controlled by a driven board (Quad-Key, Bartels mikrotecknik, Dortmund, Germany) was connected to the channel outlet and aspirates the fluid sample. This allowed to change only the flacon with the solution at the inlet, speeding up the experimental campaigns’ acquisitions. A halogen light source (LS-1 Tungsten Halogen Light Source, Ocean Optics, Dunedin, FL, USA), providing visible light, was used as optical actuation system. A simultaneous acquisition with two different instruments, i.e., spectrophotometer (USB2000, Ocean Optics, Dunedin, FL, USA) and photodiode (PDA100A, Thorlabs, Newton, NJ, USA), was implemented. The spectrophotometer was connected via a USB cable to a PC to collect the transmission measurements through the Spectra Suite 2.0 dedicated software.

In $Setup_{2}$, syringe pumps (neMESYS) were used to inject the sample of microparticles suspended in a fluid into the *MoF chamber* device. A laser system (NovaPro 660-125, RGB Lasersystem, Kelhein, Germany) with an emission wavelength of 660 nm and an input power of 1 mW was used as optical actuation system. Here, both $OF_{1}$ and $OF_{2}$ were connected to a photodiode. A microscope (B-380, OPTIKA, Ponteranica, BG, Italy), including the hardware components used to retain and align the optical elements and the device, was used with a magnification lens of 4× (PLN, Olympus, Tokyo, Japan) to scale up the channel images and increase the image resolution. A CCD camera (340M Fast Frame, Thorlabs) with a resolution of 640×480 px (pixel size of 7.4 μm, square), coupled with the microscope, was connected through a USB cable to a PC for the data acquisition in the dedicated ThorCam™ (v.3.7.0) Software.

In both described experimental setups, photodiodes were set with a gain of 40 dB and the signals received were acquired using an PC oscilloscope (Picoscope 2204A, Pico Technology, Cambridgeshire, UK), with a sampling frequency of 1.5 kHz. Acquisitions and analyzes were performed using a PC with an Intel Core i7 processor, INTEL Iris Xe Graphics, 16 GB RAM, and a 512 GB SSD.

## 5. Fluids Characterization

### 5.1. Experimental Campaign

With the aim of proving the *MoF chamber*’s suitability for differentiating between fluids of various colors and properties through optical detection techniques, different categories of fluids were investigated in two sets (*Set-1* and *Set-2*) for a total of 10 experiments.

- *Set-1*: investigates samples of colored water in different shades. More specifically: *yellow*, *red*, *green*, and *blue* water;
- *Set-2*: analyzes fluids at different densities and refractive index values. More precisely: *air*, *water*, *PBS*, *water–glycerol 16% (Gl. 16%)*, *water–glycerol 33% (Gl. 33%)* and *water–glycerol 80% (Gl. 80%)*. Their density and refractive indexes are reported in Table 2.

For each condition, the investigated fluid was delivered inside the *MoF chamber* at a constant flow rate (*A* = 0.01 mL/min) and a 60 s acquisition was carried out.

### 5.2. Results and Discussion

This section provides the results of the fluid characterization that demonstrate the features of the optical detection integration. In particular, the spectrophotometer is shown to be suitable to discriminate colored fluids thanks to its sensitivity to different wavelengths. On the other hand, optical detection performed through photodiodes is particularly useful to discriminate between fluids with different properties, such as density and refractive index. After the simultaneous acquisition of the signals through $Setup_{1}$ (see Figure 7b), the optical signals acquired through the photodiodes were subjected to a signal post-processing phase, where a low-pass filter with a 40 Hz cutoff frequency was applied to eliminate high-frequency components.

**Figure 8:**
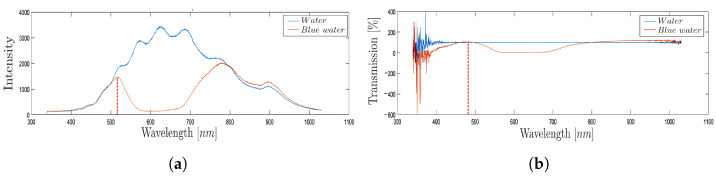
(**a**) Trends and (**b**) transmission percentage obtained through the spectrophotometer acquisition during the passage of blue-colored water in the *MoF chamber* with water as a reference.

As an example, Figure 8 shows the intensity (Figure 8a) and the transmission percentage (Figure 8b) obtained through the spectrophotometer acquisition during the passage of blue-colored water in the *MoF chamber* (orange line) and the water imposed as reference (blue line). The signals obtained are time-independent and represent a static acquisition of the process carried out within the first 5 s of fluid flow inside the *MoF chamber*. By looking at the acquired signals through the spectrophotometer (orange lines) in Figure 8a,b, the transmission peak appears at about 500 nm, which is the theoretical wavelength of blue. This exemplifies how the spectrophotometer’s measurement of transmission is suitable to discriminate which colored water liquid is present inside the *MoF chamber* thanks to the different wavelength at which fluids are most sensitive.

**Figure 9:**
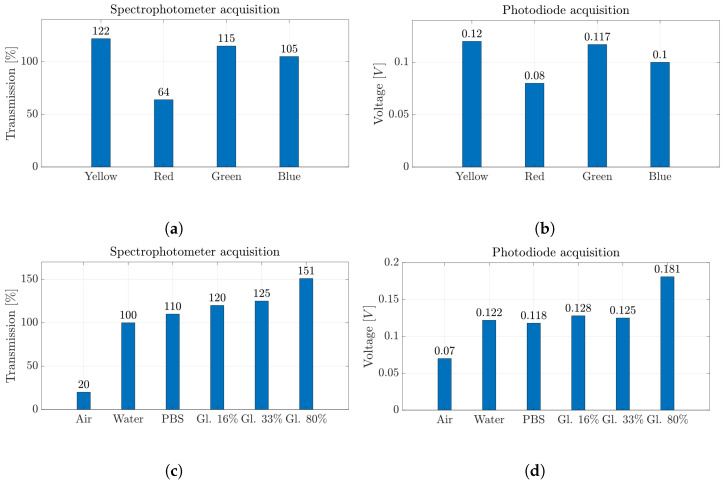
(**a**,**c**) Percentage values of transmission obtained by spectrophotometer acquisition and (**b**,**d**) voltage values obtained by photodiode acquisition for all the fluids investigated in the micro-optofluidic *MoF chamber*.

In Figure 9a,b, the results of the measurements acquired with the two instruments for *Set-1* are summarized. In particular, the maximum transmission values in the visible electromagnetic spectrum (Figure 9a) and the average voltage values of the signals acquired with the photodiode (Figure 9b) show the same trends. Moreover, the wavelength obtained for each color belongs to the corresponding value, i.e., red (625–740 nm), blue (435–500 nm), green (520–565 nm) and yellow (565–590 nm) wavelength bands.

In contrast, Figure 9c,d report the average transmission values and the average voltage values for the different fluids investigated in *Set-2*. It is worth noting that as the fluid’s refractive index rises, both devices record higher values. Thus, there exist increasing trends in Figure 9c,d.

## 6. Microparticle and Velocity Detection

In this section, we investigate the effects of employing a solution containing microparticles in suspension rather than a fluid-only solution to fill the *MoF chamber*. More precisely, the ability of the device to initially identify the presence of microparticles using optical detection systems is investigated. After testing the capacity of the device for this purpose, the silica beads’ dynamic behavior was analyzed by estimating their velocity through our DPSV methodology. A comparative study between the latter and the DPIV-based algorithm was performed as proof of effectiveness.

### 6.1. Experimental Campaign

Polymethyl methacrylate (PMMA) silica beads having a diameter of 6 μm and a density value of 1.2 g/cm${}^{3}$ were investigated. Two set of experiments were carried out, *Set-1* and *Set-2*.

In *Set-1*, an experimental campaign was carried out by using $Setup_{1}$ to evaluate the device’s ability to detect the presence of microparticles when the *MoF chamber* is filled with a solution containing microparticles in suspension. For that purpose, silica beads suspended in two fluids were tested. Phospate-buffered saline (PBS) was selected as the suspension fluid, while a water–glycerol (Gl. 80%) solution obtained by combining water and glycerol at a mixing ratio of (20:80), was used to prevent the sinking of the microparticles to the bottom of the channel, thanks to their comparable density values. In this set of experiments, solutions were injected inside the *MoF chamber* at a fixed flow rate *A* = 0.01 mL/min.

In *Set-2*, an experimental campaign was conducted with the aim of showing the effectiveness of the DPSV methodology proposed here in estimating the microparticle velocity inside the *MoF chamber*. For that purpose, silica beads were diluted in 10 mL of water–glycerol solution (Gl. 80%). The samples were fed into the *MoF chamber* using oscillating flows at different frequency (*f*) and different amplitude values (*A*). To test the performance of the proposed device in different experimental contexts, two input flow rate conditions were considered: continuous feeding of the sample in the *MoF chamber* and pulsatile feeding. A schematic summary of the performed experiments is presented in Table 3.

### 6.2. Results and Discussion

Initially, the results related to the capability of microparticle detection are presented, considering $Setup_{1}$, where both spectrophotometer and photodiode acquisitions were considered. Then, the possibility of detecting both the microparticles’ presence and their velocity was proven using $Setup_{2}$ and the DPSV methodology. The novel DPSV method was used to analyze the data collected in the experimental campaign in *Set-2* according to the method described in Section 3.2. Then, the velocity values $V_{DPSV}$ were compared with $V_{DPIV}$ obtained through the DPIV-based algorithm described in Section 3.1. The performance of the DPSV method proposed was discussed in both continuous and pulsatile input flow rate operative conditions.

#### 6.2.1. Microparticle Detection

**Figure 10:**
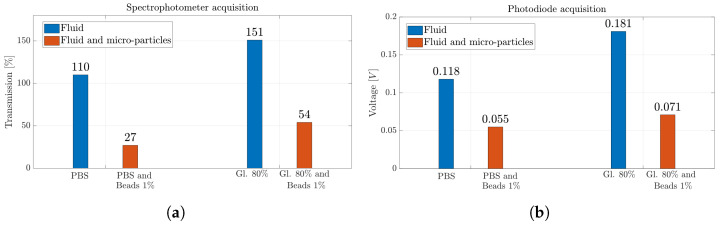
(**a**) Percentage value of transmission obtained with spectrophotometer acquisition and (**b**) voltage value obtained with photodiode acquisition for solutions of silica beads in suspension.

Figure 10a,b show the results obtained for the silica beads suspended in PBS and Gl. 80% solutions with the two different measurement systems, i.e., spectrophotometer and photodiode. While the latter is able to identify the presence of silica beads in the solutions distinguishing between whether the *MoF chamber* is filled with fluids or solutions containing suspended microparticles, the spectrophotometer shows greater sensitivity in detecting the presence of microparticles when suspended in a PBS solution. Indeed, there is a reduction of 75% in the transmission value observed between PBS and PBS with silica beads in suspension. In contrast, the observed drop in percentage is about 53% when comparing the values obtained from the photodiode acquisition. Similar percentage decreases are obtained when the Gl. 80% solution is considered. They are equal to 64% and 61% considering the spectrophotometer and the photodiode detection system, respectively.

#### 6.2.2. Continuous Input Flow Rate Condition

**Figure 11:**
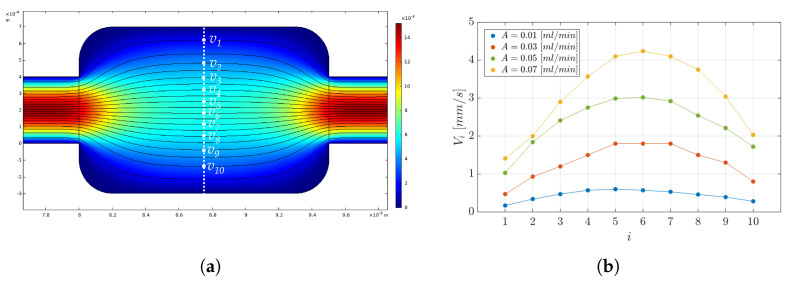
(**a**) Velocity field distribution and velocity streamlines in the experimental conditions with A=0.01 mL/min. The white dashed line indicates the region where the velocity values $v_{i}$ mm/s with i=1,...,10 were considered (white dots) to calculate the mean velocity in the *MoF chamber* from the CFD analysis ($V_{Simulated}$). (**b**) Parabolic velocity profiles in the chosen region in the experimental conditions with A∈{0.01,0.03,0.5,0.07} mL/min.

As a benchmark of the experimental results obtained, a set of computational fluid dynamics (CFD) analyses was run to evaluate the fluid velocity field distribution, as a microparticle carrier, and the velocity streamlines. The CFD setting consisted of: (i) definition of the geometry of the *MoF chamber*, (ii) specification of surface material (PDMS) and fluid (water–glycerol solution (Gl. 80%)), (iii) choice of the physical phenomenon of laminar flow with continuous input flow, (iv) triangular normal mesh physics-controlled, and (v) stationary study. Figure 11a shows the velocity field distribution and the velocity streamlines in the experimental conditions with *A* = 0.01 mL/min. In detail, a set of measurements were conducted in a region of the *MoF chamber* characterized by the higher level of variation, the one indicated by the white dashed line in Figure 11a, to estimate the average velocity in a central region of the *MoF chamber*. Different velocity values $v_{i}$ mm/s, with i=1,...,10, were extracted from the streamlines (white dots) and used to calculate the mean velocity ($V_{Simulated}$) in the *MoF chamber* and the associated standard error in a set of 10 measurements. Figure 11b reports the 10 velocity measurements for A∈{0.01,0.03,0.5,0.07} mL/min. The parabolic profile in the *MoF chamber* is shown for each value of *A*, referring to a flow velocity distribution in which the fluid velocity increases when moving from the center of the microchannel towards the walls. Notably, the higher the value of *A*, the higher the variation between the $max(v_{i})$ and the $min(v_{i})$. In summary, increasing the input flow rate in a microchannel results in a more pronounced parabolic velocity profile with higher velocities at the channel centerline. This is a consequence of the increased pressure-driven force and the balance between pressure and viscous forces in the microchannel, as described by Poiseuille flow [66].

**Figure 12:**
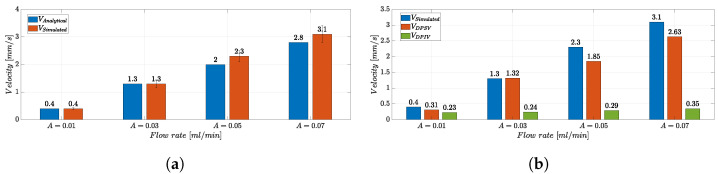
(**a**) Bar plot of the velocity values $V_{Analytical}$ and $V_{Simulated}$ in the experimental conditions with *f* = 0 Hz and A∈{0.01,0.03,0.5,0.07} mL/min. For the parameter $V_{Simulated}$, the error bar, calculated as the standard error in i=1,...,10 measurements, is reported. (**b**) Bar plot of the velocity values $V_{Simulated}$, $V_{DPSV}$, and $V_{DPIV}$ in the experimental conditions with *f* = 0 Hz and A∈{0.01,0.03,0.5,0.07} mL/min.

Figure 12a shows the comparison between the $V_{Simulated}$ and the analytical velocity values $V_{Analytical}$, obtained by relying on theoretical considerations for the input flow rate *A* and the *MoF chamber* section *S* = 400 μm × 1 mm, according to Equation (5):

(5) $$V_{Analytical}=\frac{A\;[\mathrm{mL}/\min]}{60\;\left[s\right]\times S\;\left[{\mathrm{mm}}^{2}\right]}$$

As a result, the average velocity value in the *MoF chamber* estimated through a set of measurements in the CFD analysis ($V_{Simulated}$) is consistent with the analytical one ($V_{Analytical}$). Additionally, the Reynolds numbers in the microchannel and in the *MoF chamber* were calculated, resulting in Re∈{0.0004,0.0012,0.002,0.0028} and Re∈{0.00023,0.00075,0.0012,0.0016}, for A∈{0.01,0.03,0.5,0.07} mL/min respectively, indicating that the flow is in the laminar regime.

Figure 12b shows the bar plot of the velocity values $V_{Simulated}$, $V_{DPSV}$, and $V_{DPIV}$ in the experimental conditions with *f* = 0 Hz and A∈{0.01,0.03,0.5,0.07} mL/min. The validity of the DPSV method in the microparticle’s velocity estimation is verified by $V_{DPSV}$, very close to $V_{Simulated}$. On the other hand, $V_{DPIV}$ values are saturated to a very low velocity level. Indeed, the DPSV approach is proven to be robust and suitable for fast dynamic flow manipulation and control. In fact, as the velocity of the microparticles rises, the DPIV-based algorithm’s capability to estimate a velocity value consistent with the expected value analytically decreases. This is justified by the limited camera frame rate that restricts the upper bound of the measurable velocity range, introducing significant motion blur to the particle images when their velocity becomes higher [67].

#### 6.2.3. Pulsatile Input Flow Rate Condition

The analysis that allows to investigate the effect that can be induced in the process by changing the frequency *f* and the amplitude *A* of the external oscillating input flow strength, detailed in the experimental campaign (*Set-2*), is reported below. When the input flow rate is oscillatory, the time needed to reach the maximum (to overcome the transitory phase) is greater than the oscillatory time. As a consequence, a difference of two orders between the stream velocity and the particles’ velocity is obtained [47], since the effect of the drag force cannot be extinguished.

**Figure 13:**
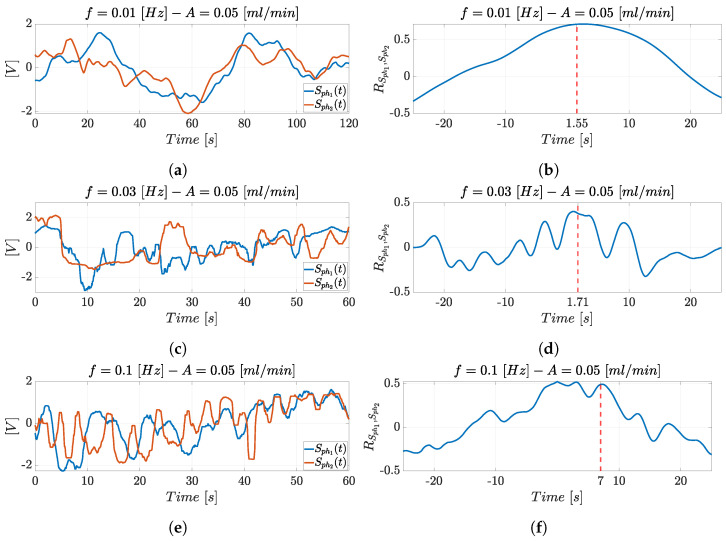
Superimposition of the photodiodes’ optical signals trends $S_{ph_{1}}\left(t\right)$ and $S_{ph_{2}}\left(t\right)$ and their cross-correlation $R_{S_{ph_{1}},S_{ph_{2}}}$ in the experimental condition with A=0.05 mL/min and (**a**,**b**) f=0.01 Hz, (**c**,**d**) f=0.03 Hz, and (**e**,**f**) f=0.1 Hz.

Figure 13 reports the superimposition of the photodiodes’ optical signals trend $S_{ph_{1}}\left(t\right)$ and $S_{ph_{2}}\left(t\right)$ (left) and their cross-correlation $R_{S_{ph_{1}},S_{ph_{2}}}$ (right) in the experimental condition with A=0.05 mL/min and f=0.01 Hz (Figure 13a), f=0.03 Hz (Figure 13c), and f=0.1 Hz (Figure 13e). The oscillation period of the optical signals decreases when moving from Figure 13a to Figure 13c,e. In terms of fluid dynamics, the particles’ displacement in a fluid is affected by the drag force that increases with the variation in density and velocity. Therefore, higher frequencies cause more rapid changes in the particles’ displacement in comparison to lower frequencies, resulting in less regular flow. This is reflected in the noisy signals detected at higher frequency.

**Figure 14:**
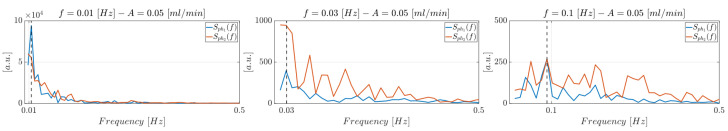
Superimposition of the photodiodes’ optical signals spectra $S_{ph_{1}}\left(f\right)$ and $S_{ph_{2}}\left(f\right)$ in the experimental condition with A=0.05 mL/min and f=0.01 Hz (**left**), f=0.03 Hz (**center**), and f=0.1 Hz (**right**).

The input hydrodynamic actuation system’s periodicity can be observed in the optical signals, as well as in the maximum frequency peak in the spectra reported in Figure 14. Moreover, each graph shows that the two optical signals shifted in time over a time interval which is the time value (Δt) of interest provided by the cross-correlation to estimate microparticle velocity. The cross-correlation trends $R_{S_{ph_{1}},S_{ph_{2}}}$ and the maximum peak detected at the corresponding delay Δt (in red) are shown in the experimental condition with A=0.05 mL/min and f=0.01 Hz (Figure 13b), f=0.03 Hz (Figure 13d), and f=0.1 Hz (Figure 13f). The Δt detection allows to estimate the microparticles’ velocity starting from the cross-correlation function, following the procedure reported in Section 3.1.

**Figure 15:**
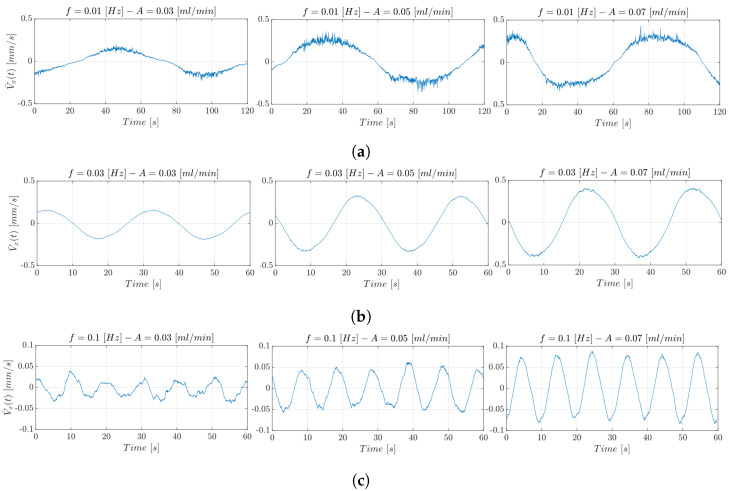
${\bar{V}}_{x}\left(t\right)$ estimated through the DPIV-based algorithm in the experimental conditions with (**a**) f=0.01 Hz, (**b**) f=0.03 Hz, (**c**) f=0.1 Hz, and A∈0.03,0.05,0.07 mL/min.

Using the pulsatile flow rate, the microparticle velocity values obtained are lower that 0.5 mm/s, so the DPIV-based approach represents a valid benchmark. Figure 15 shows the trend of the ${\bar{V}}_{x}\left(t\right)$ estimated through the DPIV-based algorithm. In particular, from left to right in the figures, it is possible to observe the microparticles’ hydrodynamic response at constant *f* and increasing amplitude, i.e., A∈{0.03,0.05,0.07}.

In the three figures, as the amplitude *A* of the oscillatory input flow grows, the maximum speed reached by the microparticles rises. For instance, the velocity increases from 0.02 mm/s up to 0.04 mm/s when the input oscillating frequency *f* is set equal to 0.01 Hz. Similar trends are obtained for the other investigated oscillatory frequency values. In Figure 15, the relationship between frequency and velocity excursion is illustrated. In particular, from (a) to (c), it can be observed that as the *f* increases, the velocity excursion decreases. The lower the input frequency, i.e., f=0.01 Hz, the higher the signal range, since a slower input flow can be propagated more efficiently.

**Figure 16:**
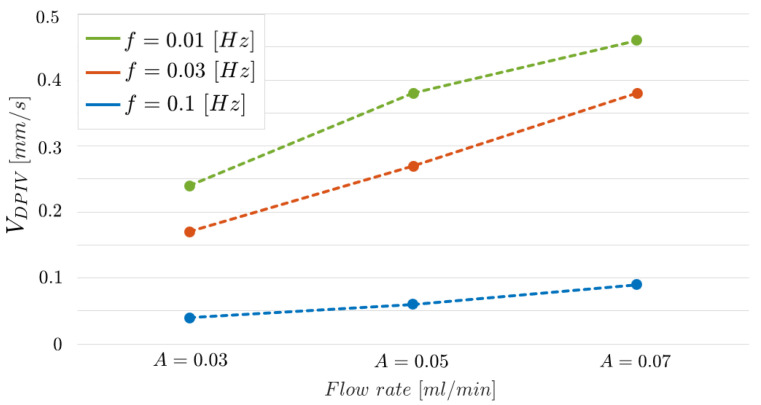
$V_{DPIV}$ parameter estimated using the DPIV-based algorithm varying the input flow rate A∈{0.03,0.05,0.07}. Each curve is related to an input oscillating frequency (*f*) value.

Figure 16 shows further evidence of such a result. The three curves, one for each frequency value, report $V_{DPIV}$ obtained by varying the input flow rate A∈{0.03,0.05,0.07}. The curve related to f=0.03 Hz is arranged in an intermediate position between the two curves but near the one associated to f=0.01 Hz, as it is expected since the two frequency values are closer.

**Figure 17:**
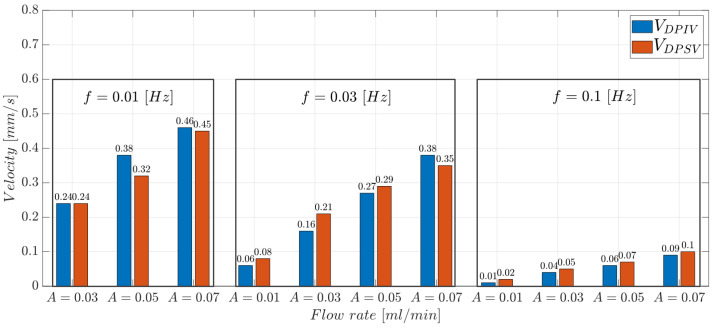
$V_{DPIV}$ and $V_{DPSV}$ parameters varying the input flow rate A∈{0.01,0.03,0.05,0.07}. Each box is related to an input oscillating frequency (*f*) value.

The values experimentally collected with the DPSV approach were compared with those obtained with the DPIV-based algorithm. The bar plots in Figure 17 report the comparison of the $V_{DPIV}$ and $V_{DPSV}$ values obtained through the two methods. In the experimental condition with f=0.01 Hz there is a percentage decrease of about 15% (A=0.07 mL/min) and 2% (A=0.05 mL/min) between $V_{DPIV}$ and $V_{DPSV}$. Similar results were obtained at f=0.03 Hz and f=0.01 Hz. Note that the slower the input flow, the more efficient the input propagation, due to the lower microparticle flow inertia. This guarantees optical signals that better follow the hydrodynamic response of microparticles. Thus, DPSV provided velocity values that fit with those estimated using the DPIV-based algorithm, confirming the feasibility of the proposed methodology.

## 7. Conclusions

This work presents a multi-objective micro-optofluidic device, called an *MoF chamber*, able to analyze fluids and microparticles in suspension using non-invasive optical detection techniques, offering the opportunity to use different optical acquisition. The cost-effective fabrication protocol selected for the realization of the *MoF chamber*, i.e., the master–slave approach based on an inkjet 3D-printing technology, allowed to manufacture the designed device with a time-saving and safe procedure. Furthermore, the fabrication protocol proposed in this paper allowed for the production of a final MoF device showing any issue related to its quality, both in terms of surface roughness and size accuracy. In fact, the latter issue was avoided by proceeding with a DfAM strategy which permitted the acquisition of the designed nominal values for the fundamental size determined in simulation phase.

A novel, easy, and cost-effective dual-slit particle signal velocimetry (DPSV) method was proposed and validated using suspended silica bead solutions. Its potentials and performance on detecting microparticle velocity were compared to numerical approach and experimental approach based on image detection, i.e., the DPIV-based algorithm, proving its effectiveness with the significant advantages of not requiring a CCD camera and an almost complete reduction in computational time. The latter benefit together with the suitability of micro-optical components for integration, make the combination of the device and the proposed DPSV method a proof of concept towards the challenge of real-time total-on-chip analysis, for the real-time monitoring and control of biological samples or chemical reaction in the chamber. In addition, the possibility of parallelizing the image acquisition through CCD images and signal acquisition via photodetector can pave the way for simultaneous process control via signal processing and process spatio-temporal monitoring using imaging techniques.

In future works, one possibility could be to consider micro-optical components integrated directly into the device in view of the development of low-cost and portable devices. Moreover, the proposed methodology could be extended to higher dimension microparticles as well as biological solutions and will be optimized to be suitable for real-time detection in embedded systems and the development of standalone platforms Additionally, it could be considered to load the chip with a fluorescent solution to visualize the projection of light through the *MoF chamber* and better estimate where the light is going, as an additional test to eventually optimize the optical detection since our goal is to develop a label-free system that does not use dyes.

## Figures and Tables

**Table 1:** Dimensions of the prototypes D-1, D-2 and D-3.

	MoF Chamber Dimensions (Length×Width×Height)	Fiber Insertion Width ($w_{\mathrm{IF}}$)	Optical Fiber Distance ($d_{i}$)
**D-1**	1 mm × 1 mm × 1 mm	1 mm	0.5 mm
**D-2**	1 mm × 1.5 mm × 0.4 mm	0.4 mm	0.5 mm
**D-3**	1 mm × 1.5 mm × 0.4 mm	0.4 mm	1 mm

**Table 2:** Density and refractive index values for the fluids investigated.

Fluids	Density g/cm${}^{3}$	Refractive Index
Air	-	1
Water	1	1.33
Phosphate-buffered Saline	1.072	1.34
Water–glycerol 16% (Gl. 16%)	1.035	1.47
Water–glycerol 33% (Gl. 33%)	1.074	1.47
Water–glycerol 80% (Gl. 80%)	1.2	1.47

**Table 3:** Calibration phase and microparticle velocity detection experimental campaign.

	Microparticles	Frequency *f* [Hz]	Amplitude *A* [mL/min]
*Set-1*	PBS and Beads 1%	-	0.01
	Gl. (80%) and Beads 1%	-	0.01
*Set-2*	Gl. (80%) with 1% of beads	-	0.01, 0.03, 0.05, 0.07
		0.01	0.03, 0.05, 0.07
		0.03	0.01, 0.03, 0.05, 0.07
		0.1	0.01, 0.03, 0.05, 0.07

## Data Availability

Data will be made available on request.
